# Identity Functioning and Eating Disorder Symptomatology: The Role of Cognitive Emotion Regulation Strategies

**DOI:** 10.3389/fpsyg.2021.667235

**Published:** 2021-05-28

**Authors:** Margaux Verschueren, Laurence Claes, Nina Palmeroni, Leni Raemen, Tinne Buelens, Philip Moons, Koen Luyckx

**Affiliations:** ^1^Department of Psychology, KU Leuven, Leuven, Belgium; ^2^Faculty of Medicine and Health Sciences, University of Antwerp, Antwerp, Belgium; ^3^Department of Paediatrics and Child Health, Faculty of Health Sciences, University of Cape Town, Cape Town, South Africa; ^4^Department of Public Health and Primary Care, KU Leuven, Leuven, Belgium; ^5^Unit for Professional Training and Service in the Behavioural Sciences, University of the Free State, Bloemfontein, South Africa

**Keywords:** eating disorders, identity development, emotion regulation, adolescence, cross-lagged

## Abstract

**Introduction:** Adolescence is the most critical life period for the development of eating disorder (ED) symptomatology. Although problems in identity functioning and emotion dysregulation have been proven important risk and maintaining factors of ED symptomatology, they have never been integrated in a longitudinal study.

**Methods:** The present study is part of the Longitudinal Identity research in Adolescence (LIA)-study and aimed to uncover the temporal interplay between identity functioning, cognitive emotion regulation, and ED symptomatology in adolescence. A total of 2,162 community adolescents (Time 1: 54% female; *M*_age_ = 14.58, *SD* = 1.88, range = 10–21 years) participated at three measurement points with 1-year intervals. They reported on identity functioning (identity synthesis and identity confusion), cognitive emotion regulation (rumination, catastrophizing, and positive reappraisal), and ED symptomatology (drive for thinness and bulimia symptoms).

**Results:** Cross-lagged paths could be fixed for boys and girls and showed bidirectional associations between both dimensions of identity functioning and both rumination and catastrophizing over time. Similarly, these maladaptive cognitive emotion regulation strategies were bidirectionally related to ED symptomatology over time. Finally, indirect pathways pointed to bidirectional associations between both dimensions of identity functioning and bulimia symptoms through rumination and catastrophizing. Only unidirectional associations emerged for drive for thinness and almost no cross-lagged associations were found with positive reappraisal.

**Conclusion:** The present study demonstrates that identity confusion may contribute to the development of ED symptomatology in adolescence through cognitive emotion dysregulation. It also reveals that these ED symptoms hamper identity development through emotion dysregulation. These results stress the importance of targeting both identity functioning and cognitive emotion regulation in the prevention and intervention of ED symptoms.

## Introduction

Disturbed eating behavior is highly prevalent during adolescence (Croll et al., 2002; Neumark-Sztainer et al., 2011)—a life period characterized by significant body changes (Susman et al., 2010). For adolescents, and especially for girls, the combination of weight gain (typical for adolescence) and being confronted with daily images of “perfect” thin and slender bodies (e.g., through social media) can become highly distressing and may result in a drive for thinness and disturbed eating behaviors (Thompson et al., 1999; Jones et al., 2004). Adolescents who experience greater identity confusion—lacking a sense of self-continuity and purpose in life (Erikson, 1968)—seem particularly susceptible to internalize such elusive beauty ideals and to experience body dissatisfaction (Vartanian et al., 2018). Existing research has indeed demonstrated the critical role of identity functioning in eating disorder (ED) symptomatology (Schupak-Neuberg and Nemeroff, 1993; Verstuyf et al., 2014; Verschueren et al., 2018).

Identity confusion is generally accompanied by lower self-esteem and increased internalizing symptoms (Schwartz et al., 2009), which can be regulated in both adaptive and maladaptive ways. Especially in adolescence, the heightened experience of negative affect is not yet met by fully matured emotion regulation capacities, resulting in an increase of maladaptive strategies (Zimmermann and Iwanski, 2014; Cracco et al., 2017). As such, they experiment with various strategies to deal with negative emotions—possibly elicited by identity confusion—and some may turn to dieting, binge eating, and purging behavior (Overton et al., 2005; Corstorphine, 2006; Smyth et al., 2007). However, although research has pointed to the prospective relation between identity functioning and ED symptomatology (Verschueren et al., 2018), very little is known about the role of emotion regulation in this relationship. Hence, the purpose of the present longitudinal study is to explore the temporal associations linking identity functioning, emotion regulation, and ED symptomatology in adolescence.

### Identity Functioning and ED Symptomatology

In early adolescence, boys and girls generally start questioning who they are and what they want to achieve in life. Erikson (1968) described this search as an identity crisis, in which individuals aim to reach a sense of continuity and sameness over time and situations. If such *identity synthesis* is reached, they are able to integrate childhood identifications with newly made commitments in life (Erikson, 1968). However, throughout adolescence, some degree of *identity confusion* can emerge (Erikson, 1968). Identity confusion is characterized by uncertainty about identity-related questions in various domains, such as long-term goals, career choices, friendships, religion, values or beliefs, etc. (Côté and Levine, 1987; Berman et al., 2004). This generally causes some distress, as the adolescent may feel somewhat lost in this identity quest and can feel overwhelmed by all available options. Moreover, they often experience societal pressures to resolve these questions quite quickly (Erikson, 1968). Research on identity functioning in adolescence and emerging adulthood indeed indicates that individuals who lack identity commitments (i.e., indicative of identity confusion; Schwartz et al., 2005), score higher on depressive symptoms and anxiety compared to individuals who are committed to life choices (Luyckx et al., 2008; Crocetti et al., 2011, 2012).

#### From Identity to ED Symptomatology

Previous research has established a clear association between identity functioning and ED symptomatology in both boys and girls. Studies in clinical samples show that patients with an ED generally experience more identity confusion compared to individuals in control samples (Schupak-Neuberg and Nemeroff, 1993; Sparks, 1993). Issues in identity development are described as core deficits of patients with an ED and are regarded as one of the causing mechanisms of ED symptomatology (Bruch, 1981, 1982; Heatherton and Baumeister, 1991; Schupak-Neuberg and Nemeroff, 1993; Fairburn et al., 2003). In recent years, there has been an increasing amount of research in community samples as well. Vartanian (2009) and Verstuyf et al. (2014) found that individuals with lower self-concept clarity (similar to identity confusion) and individuals who want to conform to expectations from others in constructing their identity (i.e., normative identity style; Berzonsky, 2004) are more susceptible to internalize body ideals. Such ideals may stimulate thin and muscular bodies for women and men, respectively (Lawler and Nixon, 2011). Hence, especially for women, a drive for thinness may develop and may even become a (maladaptive) source of self-definition (Polivy and Herman, 2007). Taken together, ED symptomatology can be induced by distress from experiencing identity confusion and can also represent a new way to define oneself (Polivy and Herman, 2007).

#### From ED Symptomatology to Identity

A number of studies have also begun to examine the opposite direction—how ED symptomatology can hamper identity functioning (Verschueren et al., 2020a). Various researchers (Fairburn et al., 2003; Corning and Heibel, 2016; Klimstra and Denissen, 2017) have described how pathological ED cognitions and behaviors can become an important part of one's identity content, as—in individuals with disturbed eating behaviors—eating habits and body shape often become one of the main sources of self-definition and self-worth (Fairburn et al., 2003; Corning and Heibel, 2016). Moreover, constantly focusing on eating behavior and body shape may hamper important other identity domains, such as engaging in relationships with others or school/work adjustment and performance. Consequently, such alternative identity contents may have decreased opportunities to come to fruition (Corning and Heibel, 2016). Consequently, the individual does not acquire a coherent whole of identity domains that fit together, generally resulting in identity confusion (Erikson, 1968). ED symptomatology can thus hamper constructive identity work and derail a normative identity development (Verschueren et al., 2020a).

#### Temporal Interplay of Identity and ED Symptomatology

A recent study in community adolescents (Verschueren et al., 2018) investigated the temporal interplay between identity functioning and ED symptomatology. Identity confusion predicted a relative increase in body dissatisfaction and bulimia symptoms over a 1-year period, whereas these symptoms also predicted a relative increase in identity confusion over time. Similar findings occurred for identity synthesis as it seemed to protect against the development of body dissatisfaction, bulimia, and drive for thinness. Conversely, body dissatisfaction and bulimia symptoms negatively predicted identity synthesis over time. These prospective associations were identical in early to late adolescents and across gender. Hence, the results from this previous study support the hypothesized bidirectional relation between identity functioning and ED symptomatology.

### Emotion Regulation and ED Symptomatology

Emotion regulation has been described as the process of regulating the experience and expression of emotions that emerge to everyday events (Gross and Thompson, 2007). Kraaij and Garnefski (2019) have distinguished cognitive emotion regulation (i.e., attempting to regulate emotions by changing cognitions) from behavioral emotion regulation (i.e., attempting to manage or change the situation with action)—representing two conceptually distinct types of emotion regulation that are used at different points in time (Garnefski et al., 2001). Especially during (middle) adolescence, emotional stability is lowest and youth experience a dysfunctional shift toward an increased use of maladaptive strategies (Zimmermann and Iwanski, 2014; Cracco et al., 2017). Emotion regulation is a transdiagnostic factor for the development and maintenance of various psychological disorders (Lukas et al., 2018). More specifically, rumination, catastrophizing, and positive reappraisal are the cognitive emotion regulation strategies that are most predictive of depressive and anxiety symptoms (Garnefski and Kraaij, 2006). Moreover, maladaptive strategies—rather than the absence of adaptive strategies—have been prospectively associated with psychopathology (Aldao et al., 2010; Aldao and Nolen-Hoeksema, 2012).

#### From Emotion Regulation to ED Symptomatology

Also with regard to EDs, emotion dysregulation is described as an important transdiagnostic risk and maintenance factor, with patients with an ED typically having trouble tolerating and effectively coping with intense moods (Fairburn et al., 2003; Svaldi et al., 2012; Brockmeyer et al., 2014; Mallorquí-Bagué et al., 2018). Also in community samples, Overton et al. (2005) have suggested that individuals who are not able to use adaptive emotion regulation strategies to handle intense emotions, may turn to eating behaviors as ways to self-regulate. More specifically, the over-controlling during restrictive eating may help the individual suppress underlying emotions, while the numbing effect of binge eating may provide relief from overwhelming negative emotions (Overton et al., 2005). In this way, disturbed eating may be regarded a behavioral emotion regulation strategy that can emerge when adaptive cognitive emotion regulation strategies are lacking. Especially young women may be vulnerable for the emotion regulatory function of eating behavior, as they are subjected to powerful cultural “thin” ideals and generally experience concerns over body image and negative affect (Overton et al., 2005). A study in female adolescents has indeed found that rumination predicts increases in bulimia symptoms over time (Nolen-Hoeksema et al., 2007).

#### From ED Symptomatology to Emotion Regulation

In their transdiagnostic theory of EDs, Fairburn et al. (2003) described ED symptomatology as “dysfunctional mood modulatory behavior,” as these symptoms may help individuals to neutralize negative emotions and cognitions, while hampering the development of healthy emotion regulation. Also Overton et al. (2005) described how the short-term benefits from engaging in ED behaviors can eventually prevent the individual from learning more adaptive emotion regulation strategies. Interestingly, especially individuals with EDs of the binge eating and purging-subtypes present with emotion regulation deficits (Mallorquí-Bagué et al., 2018; Weinbach et al., 2018). Binge eating and purging behaviors have been particularly related to emotion dysregulation as these behaviors seem to reduce the intensity of negative emotions by providing the individual with short-term comfort and distraction (Smyth et al., 2007; Mallorquí-Bagué et al., 2018). The loss of control during a binge-eating episode can create an escape from self-awareness, in which general distress can be (temporarily) avoided (Heatherton and Baumeister, 1991). Such escape-theories about binge eating and purging behavior show how these behaviors can momentarily block out emotions that the individual fails to tolerate (Cooper et al., 2004; Corstorphine, 2006). Also with regard to cognitive emotion regulation strategies, these bulimic behaviors have been found to predict an increase in rumination over time in female adolescents (Nolen-Hoeksema et al., 2007). However, also restrictive eating may function as a defense strategy to avoid difficult feelings, such as guilt and shame, in the short term (Corstorphine, 2006; Schmidt and Treasure, 2006). Focusing on restrictive eating seems to help individuals alleviating unregulated distress and has been linked with problems in emotion regulation as well (Lafrance Robinson et al., 2014; Haynos et al., 2018). Hence, while research points to emotion dysregulation being an important risk and maintenance factor of ED symptomatology, disordered eating in turn seems to hamper the development of adaptive emotion regulation strategies as well.

### Identity Functioning and Emotion Regulation

Much less is known about the association between identity functioning and emotion regulation. Early theories describe identity disturbance as an emotion regulation problem, as continuously inhibiting emotions may result in numbness and emptiness, contributing to identity disturbance (Kernberg, 1975; Linehan, 1993). In line with these ideas, Jankowski (2013) posited that effective emotion regulation determines ego strength and, thus, is a prerequisite for a healthy identity development. Focusing on the reverse direction, Jørgensen (2006) described how a stable sense of identity is regarded necessary to handle cognitive and affective regulation in a flexible way. A fragmentated self can result in an inability to contain and regulate emotions, as these individuals are unable to gain distance from a situation and make reflected decisions (Fuchs, 2007). Identity disturbance has indeed been related to emotion and behavioral dysregulation in both community and clinical samples (Neacsiu et al., 2015). Hence, having a clear idea of who you are (i.e., identity synthesis) may help understand why you feel a certain way and, subsequently, how you can act on it.

Jankowski (2013) indeed found that individuals who have engaged in identity exploration and commitment seem to experience the least difficulties in emotion awareness, whereas individuals who experience the most identity rumination, seem to experience the most emotion regulation difficulties. When focusing on coping (a construct closely related to emotion regulation, representing regulatory processes in response to a stressful event; Compas et al., 2014), especially individuals scoring high on pro-active identity exploration seem to cope successfully with daily stressors and challenges, whereas individuals scoring high on identity rumination seem to avoid dealing with their problems (Berzonsky, 1992; Luyckx et al., 2012). However, the prospective relationship between emotion regulation and identity functioning still remains unclear, as only cross-sectional studies have investigated this association.

### The Present Study

**Figure 1:**
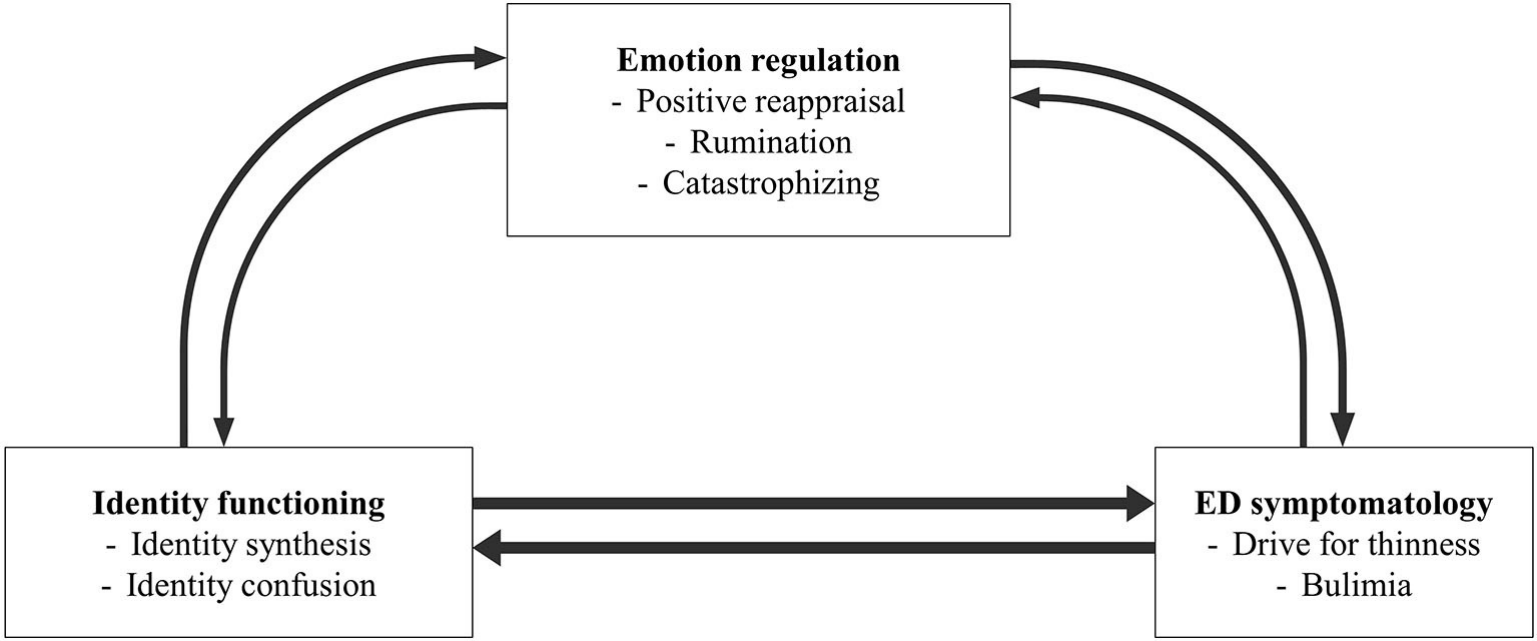
Theoretical model based on the literature.

The aim of the present study is to explore the temporal associations between identity functioning, cognitive emotion regulation, and ED symptomatology in an adolescent sample over a 2-year period. As different associations may be found between identity functioning, cognitive emotion regulation, and restrictive vs. binge-eating/purging ED symptomatology, the study includes both drive for thinness (i.e., being preoccupied with dieting and weight and the wish of being thinner; Garner, 2004) and bulimia symptoms (i.e., loss of control overeating and/or self-induced vomiting; Garner, 2004). Furthermore, the present study focuses on cognitive emotion regulation strategies, rather than behavioral strategies (Garnefski and Kraaij, 2006), as these are more conceptually distant from the well-known behavioral emotion regulation function of ED symptomatology. Doing so, both adaptive and maladaptive strategies will be included. Figure 1 is a visual representation of the interrelations among the study variables that are examined in the present study.

Based on previous research and theorizing, bidirectional associations between all study variables can be expected. With regard to the associations between identity functioning and ED symptomatology, identity synthesis is expected to predict a relative decrease in ED symptomatology, whereas identity confusion is expected to predict a relative increase in ED symptomatology over time (Verschueren et al., 2018). Similarly, ED symptomatology is expected to predict a relative increase in identity confusion and a relative decrease in identity synthesis over time. However, previous research pointed to inconsistent results for drive for thinness in this regard (Verschueren et al., 2018). The temporal association between drive for thinness and identity functioning may thus be weaker in the present study as well.

Additionally, indirect pathways from identity functioning to ED symptomatology, through cognitive emotion regulation, are expected to reach significance. Earlier theory and research already described that (1) identity confusion generally causes distress and negative emotions (Erikson, 1968; Schwartz et al., 2009) and (2) when an individual cannot regulate intense emotions, they are at risk for engaging in disturbed eating (Overton et al., 2005). More specifically, identity confusion is expected to increase vulnerability to maladaptive cognitive emotion regulation, which, in turn, would increase vulnerability to ED symptomatology over time. Similar associations would be found for identity synthesis and adaptive cognitive emotion regulation. However, whether the reverse pathway—from ED symptomatology to identity functioning, through cognitive emotion regulation—would reach significance is less clear, as no researchers have explicitly referred to such an indirect pathway. Importantly, in all models, stronger temporal associations are expected with maladaptive cognitive emotion regulation strategies, as these are generally more strongly related to psychopathology than the absence of adaptive strategies (Aldao et al., 2010; Aldao and Nolen-Hoeksema, 2012).

Inconsistent gender differences have been found with regard to identity functioning and emotion regulation. Some studies concluded that both adolescent and adult men generally present with better identity functioning than women (Verschueren et al., 2017, 2018) and other studies found opposite results (Meeus, 2011; Schwartz et al., 2011). Similarly, whereas some studies in adolescents have found women adopting more maladaptive emotion regulation strategies than men (Braet et al., 2014; Goossens et al., 2016), other studies in adults point to women generally using a broader range of (both adaptive and maladaptive) strategies than men (Tamres et al., 2002; Nolen-Hoeksema and Aldao, 2011). Hence, no clear hypotheses were made regarding gender differences in the mean levels of identity functioning and cognitive emotion regulation. However, in line with a broad range of literature (e.g., Lewinsohn et al., 2002; Abebe et al., 2012; Micali et al., 2014; Verschueren et al., 2018), girls were hypothesized to experience more drive for thinness and bulimia symptoms than boys. Finally, the temporal pathways between the study variables were expected to be equal in boys and girls, consistent with previous research (Verschueren et al., 2018).

## Materials and Methods

### Participants and Procedure

The present study is part of the Longitudinal Identity research in Adolescence (LIA)-study (Buelens et al., 2020) and was carried out at three annual measurement points (January-February 2018, January-February 2019, and January-February 2020, respectively). Eight high schools in Flanders (Belgium) participated. Beforehand, an information letter was sent out to the parents as parental consent was required for participation of minor students. The study took place during school hours and was carried out by members of the research team. Students gave informed assent for participation and put their completed questionnaires in a sealed envelope. Each student received a unique ID-code to ensure confidentiality. Students who were absent at the day of the study or who had left high school (at Times 2 and 3) were contacted via e-mail and could participate using an online web-survey to minimize drop-out. The study was approved by the ethical commission of the Faculty of Psychology and Educational Sciences of the first author.

At Time 1, 3483 high school students were invited, of which 2,313 received active parental consent (consent rate = 66.40%). Finally, 2,162 students actually participated (53.93% female; response rate = 93.5%), with a mean age of 14.58 (*SD* = 1.88, range = 10–21 years). A total of 745 participants (34.46%) were in seventh and eighth grade, while the others were in ninth to twelfth grade and followed the general track (*n* = 432; 19.98%), the technical track (*n* = 573; 26.50%), or the arts track (*n* = 412; 19.06%). At Time 2, 1,929 students participated (55.21% female; retention rate = 89.22%), with a mean age of 15.61 (*SD* = 1.83, range = 11–22 years). At Time 3, 1,751 students participated (56.25% female; retention rate = 81.00%), with a mean age of 16.57 (*SD* = 1.83, range = 12–23 years). Students who dropped out were significantly older [Time 2: *M*_retention_ = 14.60 (*SD* = 1.83), *M*_drop−out_ = 15.39 (SD = 2.13), *F*_(1,2,162)_ = 37.77, *p* < 0.001, η^2^ = 0.02; Time 3: *M*_retention_ = 14.56 (*SD* = 1.82), *M*_drop−out_ = 15.21 (SD = 2.02), *F*_(1,2,162)_ = 41.05, *p* < 0.001, η^2^ = 0.02] and more likely to be male [Time 2: %female_retention_ = 55.21%, %female_drop−out_ = 43.35%, χ^2^(1) = 11.77, *p* = 0.001, Time 3: %female_retention_ = 56.25%, %female_drop−out_ = 44.04%, χ^2^(1) = 19.99, *p* < 0.001]. However, students who dropped out did not differ significantly in identity functioning [Time 2: Wilks'∧ = 1.00, *F*_(2,2,154)_ = 0.08, *p* = 0.926; Time 3: Wilks'∧ = 1.00, *F*_(2,1,925)_ = 0.45, *p* = 0.636], ED symptomatology [Time 2: Wilks'∧ = 1.00, *F*_(2,2,155)_ = 2.33, *p* = 0.098; Time 3: Wilks'∧ = 1.00, *F*_(2,1,924)_ = 1.55, *p* = 0.212], and cognitive emotion regulation [Time 2: Wilks'∧ = 1.00, *F*_(3,2,098)_ = 0.74, *p* = 0.527; Time 3: Wilks'∧ = 1.00, *F*_(3,1,911)_ = 0.86, *p* = 0.460]. Finally, to compare the participants with and without complete longitudinal data on the study variables, Little's (1988) Missing Completely At Random (MCAR) test was conducted, indicating that missing values were not associated with the observed data of the present study [χ^2^(255) = 258.64, *p* = 0.06]. Hence, the Full Information Maximum Likelihood (FIML) procedure was used to handle such missing values.

### Measures

#### ED Symptomatology

The Eating Disorder Inventory-3 (EDI-3; Garner, 2004) is a valid and reliable screening tool for ED pathology in non-clinical populations (Van Strien and Ouwens, 2003). The present study focused on two ED Risk Scales: drive for thinness (7 items, example item: “I am preoccupied with the desire to be thinner”) and bulimia (7 items, example item “I eat moderately in front of others and stuff myself when they're gone”). All items are scored on a 6-point Likert-type scale (ranging from 1_*never* to 6_*always*). As temporal measurement invariance of all questionnaires was investigated, Confirmatory Factor Analyses (CFA) were also carried out for all questionnaires at all time points. Four fit-indices were used to examine model fit: The Satorra–Bentler scaled Chi-square (S-Bχ^2^) should be as small as possible, the Comparative Fit Index (CFI) should be > 0.90 for reasonable fit (>0.95 for excellent fit), and the Standardized Root Mean Square residual (SRMR) and the Root Mean Square Error of Approximation (RMSEA) should be close to 0.08 or below (Satorra and Bentler, 2001; Kline, 2005; Brown, 2015). CFA of the two subscales pointed to an adequate fit to the data at all time points, when allowing one error correlation between two items from the drive for thinness-subscale [Time 1: S-Bχ^2^(75) = 852.590, *p* < 0.001, CFI = 0.913, SRMR = 0.088, RMSEA = 0.069; Time 2: S-Bχ^2^(75) = 906.307, *p* < 0.001, CFI = 0.908, SRMR = 0.092, RMSEA = 0.076; Time 3: S-Bχ^2^(75) = 808.198, *p* < 0.001, CFI = 0.920, SRMR = 0.077, RMSEA = 0.075]. Additionally, longitudinal configural, metric, and scalar invariance were found for both subscales in the present study, indicating that the factor loadings and intercepts are equal over time (Van de Schoot et al., 2012; Bialosiewicz et al., 2013). Hence, such invariance allows for the cross-lagged path analyses in the present study. Information on measurement invariance of all questionnaires can be found in the Supplementary Material of this study. Finally, factor scores were exported and used in further analyses. At Time 1, 2, and 3, Cronbach's alphas for drive for thinness were 0.89, 0.92, 0.93, respectively. Cronbach's alphas for bulimia were 0.74, 0.78, 0.80, respectively.

#### Identity Functioning

The Identity Subscale of the Erikson Psychosocial Stage Inventory (EPSI; Rosenthal et al., 1981) has provided valid results to measure identity functioning in adolescents and emerging adults (Schwartz et al., 2009). The subscales identity synthesis (example item: “I've got a clear idea of what I want to be”) and identity confusion (example item: “I can't decide what I want to do with my life”) each contain six items, scored on a 5-point Likert-type scale (ranging from 1*_strongly disagree* to 5*_strongly agree*). CFA of these two subscales pointed to an adequate fit to the data at all time points [Time 1: S-Bχ^2^(53) = 538.626, *p* < 0.001, CFI = 0.908, SRMR = 0.040, RMSEA = 0.065; Time 2: S-Bχ^2^(53) = 533.031, *p* < 0.001, CFI = 0.918, SRMR = 0.039, RMSEA = 0.069; Time 3: S-Bχ^2^(53) = 497.146, *p* < 0.001, CFI = 0.926, SRMR = 0.038, RMSEA = 0.069]. Additionally, longitudinal configural, metric, scalar, and full uniqueness invariance were found for both subscales in the present study, indicating that the latent constructs are measured identically across time (Van de Schoot et al., 2012). Again, such invariance allows for the cross-lagged path analyses in the present study. Factor scores were exported and used in further analyses. At Time 1, 2, and 3, Cronbach's alphas for identity synthesis were 0.75, 0.79, 0.80, respectively. Cronbach's alphas for identity confusion were 0.67, 0.74, 0.79, respectively.

#### Cognitive Emotion Regulation

The 18-item short version of the Cognitive Emotion Regulation Questionnaire (CERQ-short; Garnefski and Kraaij, 2006) has provided reliable and valid results and distinguishes nine cognitive emotion regulation strategies in response to threatening or stressful life events in community adults and adolescents (Garnefski and Kraaij, 2006; Navarro-Loli et al., 2020). The items are scored on a 5-point Likert-type scale ranging from 1_*(almost) never* to 5_*(almost) always*. CFA of these nine subscales pointed to an excellent fit to the data at all time points [Time 1: S-Bχ^2^(99) = 557.825, *p* < 0.001, CFI = 0.952, SRMR = 0.036, RMSEA = 0.047; Time 2: S-Bχ^2^(99) = 474.331, *p* < 0.001, CFI = 0.960, SRMR = 0.031, RMSEA = 0.044; Time 3: S-Bχ^2^(99) = 539.928, *p* < 0.001, CFI = 0.949, SRMR = 0.034, RMSEA = 0.051]. Again, longitudinal configural, metric, scalar, and full uniqueness invariance were found for all nine subscales in the present study, indicating that the latent constructs are measured identically across time (Van de Schoot et al., 2012), allowing the cross-lagged path analyses in the present study. Factor scores were exported and used in further analyses. As not all nine subscales could be included in the cross-lagged models, we additionally carried out a two-factor Exploratory Factor Analysis on the nine subscales to investigate which subscales would have the highest factor loadings on an adaptive and maladaptive factor. Doing so, the present study included one adaptive strategy (i.e., positive reappraisal, λ = 0.944, example item: “I tell myself that there are worse things in life”) and two maladaptive strategies (i.e., rumination, λ = 0.967, example item: “I am preoccupied with what I think and feel about what I have experienced”; and catastrophizing, λ = 0.820, example item: “I keep thinking about how terrible it is what I have experienced”). Garnefski and Kraaij (2006) also found these three strategies to be most strongly related to depression and anxiety symptoms. At Times 1, 2, and 3, Cronbach's alphas for positive reappraisal were 0.67, 0.73, 0.73, respectively. Cronbach's alphas for rumination were 0.74, 0.75, 0.75, respectively. Cronbach's alphas for catastrophizing were 0.79, 0.82, 0.80, respectively.

### Statistical Analyses

Preliminary analyses were carried out in IBM SPSS Version 25.0. Gender differences on the latent factor scores of the study variables were examined using Multivariate Analyses of Variance (MANOVAs). Cross-lagged path analyses were conducted in Mplus (version 7.4; Muthén and Muthén, 2012) to examine the temporal sequence between the latent factor scores of identity functioning, ED symptomatology, and cognitive emotion regulation. This technique uses a structural equation modeling approach and can give an indication of temporal precedence between variables (Anderson and Kida, 1982; Kline, 2005). As longitudinal metric invariance was found for all latent variables in the present study, the cross-lagged path analyses could be carried out. Due to the high within-time correlation between the latent identity dimensions and among rumination and catastrophizing—causing multicollinearity (Kline, 2005)—their temporal relations with the other study variables were examined in separate models. To reduce the number of variables in the cross-lagged models, the temporal relations with positive reappraisal and the other study variables were examined in separate models as well. This resulted in six models, representing the directionality of effects between (Model 1) identity synthesis, ED symptomatology, and rumination; (Model 2) identity confusion, ED symptomatology, and rumination; (Model 3) identity synthesis, ED symptomatology, and catastrophizing; (Model 4) identity confusion, ED symptomatology, and catastrophizing; (Model 5) identity synthesis, ED symptomatology, and positive reappraisal; and (Model 6) identity confusion, ED symptomatology, and positive reappraisal.

In all models tested, all within-time associations, stability paths, and cross-lagged paths were examined. Due to sample size and the number of models tested, a significance level of *p* < 0.01 was used for all cross-lagged analyses. Additionally, at each time point the study variables were controlled for age and gender at baseline (i.e., allowing paths from gender and age at baseline to the study variables at each time point). Finally, the significance levels of indirect paths were checked as well. Inspired by ED theory (Schupak-Neuberg and Nemeroff, 1993; Fairburn et al., 2003; Polivy and Herman, 2007), we focused on four indirect paths in each model—provided that the cross-lagged paths in question would be significant as well: two paths going from the identity dimension at Time 1 to the ED symptoms (drive for thinness and bulimia) at Time 3 through the cognitive emotion regulation dimension at Time 2, and two paths going in the opposite direction (i.e., from the ED symptoms at Time 1 to the identity dimension at Time 3 through the cognitive emotion regulation dimension at Time 2).

First, the model fit of the baseline cross-lagged models were evaluated, using the same fit-indices as previously described (i.e., S-Bχ^2^, CFI, SRMR, and RMSEA). Second, to assess whether the cross-lagged paths were time invariant, similar cross-lagged coefficients were fixed to be equal across time (e.g., the path from identity synthesis at T1 to rumination at T2 would be constrained to be equal to the path from identity synthesis at T2 to rumination at T3). Non-significant S-Bχ^2^-differences (ΔS-Bχ^2^) indicate that the model can be constrained over time (Satorra and Bentler, 2001). Additionally, a decrease in CFI (ΔCFI) should be lower than 0.01, an increase in SRMR (ΔSRMR) should be lower than 0.01 and an increase in RMSEA (ΔRMSEA) should be lower than 0.015 (Chen, 2007). Lastly, to assess whether the cross-lagged paths were invariant for girls and boys, multi-group analyses were performed in which similar cross-lagged coefficients were fixed to be equal across gender. Again, non-significant ΔS-Bχ^2^ and values of ΔCFI and ΔSRMR lower than 0.01 and ΔRMSEA lower than 0.015 indicate that the cross-lagged coefficients can be fixed across gender.

## Results

### Preliminary Analyses

Significant gender differences were found on all latent factor scores of the study variables (Wilks'∧ = 0.77, *F*_(21,1,592)_ = 22.80, *p* < 0.001), pointing to girls experiencing more identity confusion, ED symptomatology, rumination, and catastrophizing, while reporting less identity synthesis and positive reappraisal than boys^1^. Table 1 presents all follow-up univariate *F*-values. Consistent within-time correlations were found between the latent factor scores of the study variables (see Table 2). Identity confusion was positively related to ED symptomatology and the maladaptive cognitive emotion regulation strategies, while being negatively associated to positive reappraisal. Reverse correlations were found for identity synthesis. Both drive for thinness and bulimia symptoms were positively related to the maladaptive cognitive emotion regulation strategies, whereas only drive for thinness was consistently (negatively) related to positive reappraisal.

**Table 1:** Gender Distribution of Identity Functioning, ED Symptomatology, and Emotion Regulation with Univariate ANOVA's.

**Variables**	**Time 1**			**Time 2**			**Time 3**		
	**Males *M* (*SD*)**	**Females *M* (*SD*)**	***F*_**(1, 1, 612)**_**	**Males *M* (*SD*)**	**Females *M* (*SD*)**	***F*_**(1, 1, 612)**_**	**Males *M* (*SD*)**	**Females *M* (*SD*)**	***F*_**(1, 1, 612)**_**
**Identity functioning**									
Identity synthesis	0.18 (0.47)	−0.12 (0.56)	127.82^***^	0.21 (0.50)	−0.16 (0.61)	169.77^***^	0.20 (0.61)	−0.15 (0.68)	115.05^***^
Identity confusion	−0.14 (0.37)	0.10 (0.45)	133.70^***^	−0.18 (0.43)	0.13 (0.53)	158.62^***^	−0.18 (0.52)	0.13 (0.59)	118.21^***^
**ED symptomatology**									
Drive for thinness	−0.16 (0.32)	0.14 (0.44)	243.64^***^	−0.27 (0.42)	0.22 (0.62)	328.13^***^	−0.31 (0.44)	0.24 (0.67)	367.822^***^
Bulimia	−0.16 (0.68)	0.11 (0.77)	54.91^***^	−0.20 (0.73)	0.15 (0.83)	77.67^***^	−0.24 (0.19)	0.69 (0.87)	116.43^***^
**Emotion regulation**									
Rumination	−0.25 (0.78)	0.17 (0.85)	100.16^***^	−0.23 (0.76)	0.17 (0.82)	101.85^***^	−0.22 (0.76)	0.16 (0.80)	92.16^***^
Catastrophizing	−0.22 (0.72)	0.15 (0.87)	81.40^***^	−0.22 (0.75)	0.15 (0.90)	74.63^***^	−0.20 (0.69)	0.14 (0.84)	73.48^***^
Positive reappraisal	0.05 (0.78)	−0.04 (0.80)	4.96^**^	0.08 (0.80)	−0.04 (0.81)	9.12^**^	0.04 (0.82)	−0.03 (0.81)	2.80

*A negative mean factor score represents a lower mean relative to the sample. A positive mean factor score represents a higher mean relative to the sample. ED, Eating disorder*.

** *p < 0.01*,

*** *p < 0.001*.

**Table 2:** Within-Time Correlations Among the Study Variables.

**Variables**	**2**	**3**	**4**	**5**	**6**	**7**
**Time 1 (*****N*** **=** **2,098)**						
1. Identity synthesis	−0.97^**^	−0.39^**^	−0.33^**^	−0.35^**^	−0.37^**^	0.27^**^
2. Identity confusion		0.37^**^	0.34^**^	0.39^**^	0.38^**^	−0.22^**^
3. Drive for thinness			0.41^**^	0.30^**^	0.29^**^	−0.08^**^
4. Bulimia				0.30^**^	0.29^**^	−0.02
5. Rumination					0.84^**^	0.20^**^
6. Catastrophizing						−0.08^**^
7. Positive reappraisal						
**Time 2 (*****N*** **=** **1,915)**						
1. Identity synthesis	−0.98^**^	−0.45^**^	−0.39^**^	−0.36^**^	−0.35^**^	0.28^**^
2. Identity confusion		0.44^**^	0.40^**^	0.40^**^	0.37^**^	−0.24^**^
3. Drive for thinness			0.51^**^	0.30^**^	0.29^**^	−0.09^**^
4. Bulimia				0.28^**^	0.28^**^	−0.04
5. Rumination					0.81^**^	0.21^**^
6. Catastrophizing						−0.06^*^
7. Positive reappraisal						
**Time 3 (*****N*** **=** **1,728)**						
1. Identity synthesis	−0.98^**^	−0.42^**^	−0.39^**^	−0.39^**^	−0.37^**^	0.31^**^
2. Identity confusion		0.42^**^	0.41^**^	0.41^**^	0.38^**^	−0.27^**^
3. Drive for thinness			0.63^**^	0.31^**^	0.31^**^	−0.11^**^
4. Bulimia				0.30^**^	0.29^**^	−0.07^**^
5. Rumination					0.84^**^	0.09^**^
6. Catastrophizing						−0.10^**^
7. Positive reappraisal						

* *p < 0.05*,

** *p < 0.01*.

### Temporal Relation Between Identity Functioning, ED Symptomatology, and Emotion Regulation

**Figure 2:**
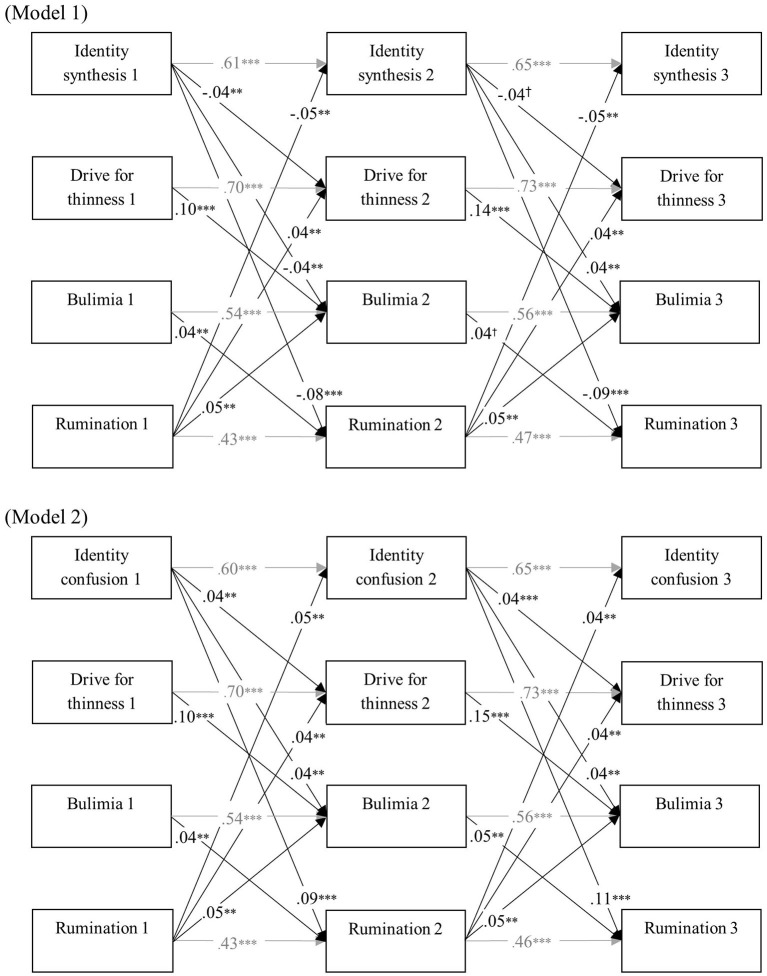
Final cross-lagged models linking identity functioning, ED symptomatology, and rumination. All models were controlled for age and gender. Within-time associations, associations with age and gender, and insignificant cross-lagged paths were not shown for reasons of clarity. All path coefficients are standardized. ^*†*^*p* < 0.05, ^**^p < 0.01, ^***^p < 0.001.

**Figure 3:**
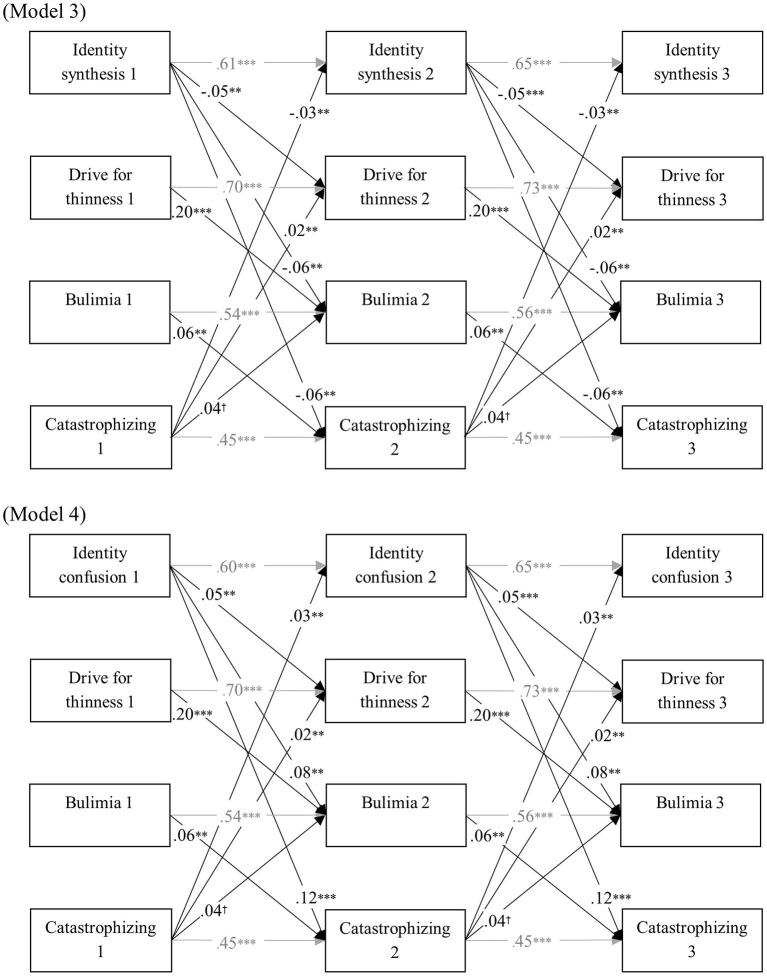
Final cross-lagged models linking identity functioning, ED symptomatology, and catastrophizing. All models were controlled for age and gender. Within-time associations, associations with age and gender, and insignificant cross-lagged paths were not shown for reasons of clarity. All path coefficients are standardized. ^*†*^*p* < 0.05, ^**^p < 0.01, ^***^p < 0.001.

**Figure 4:**
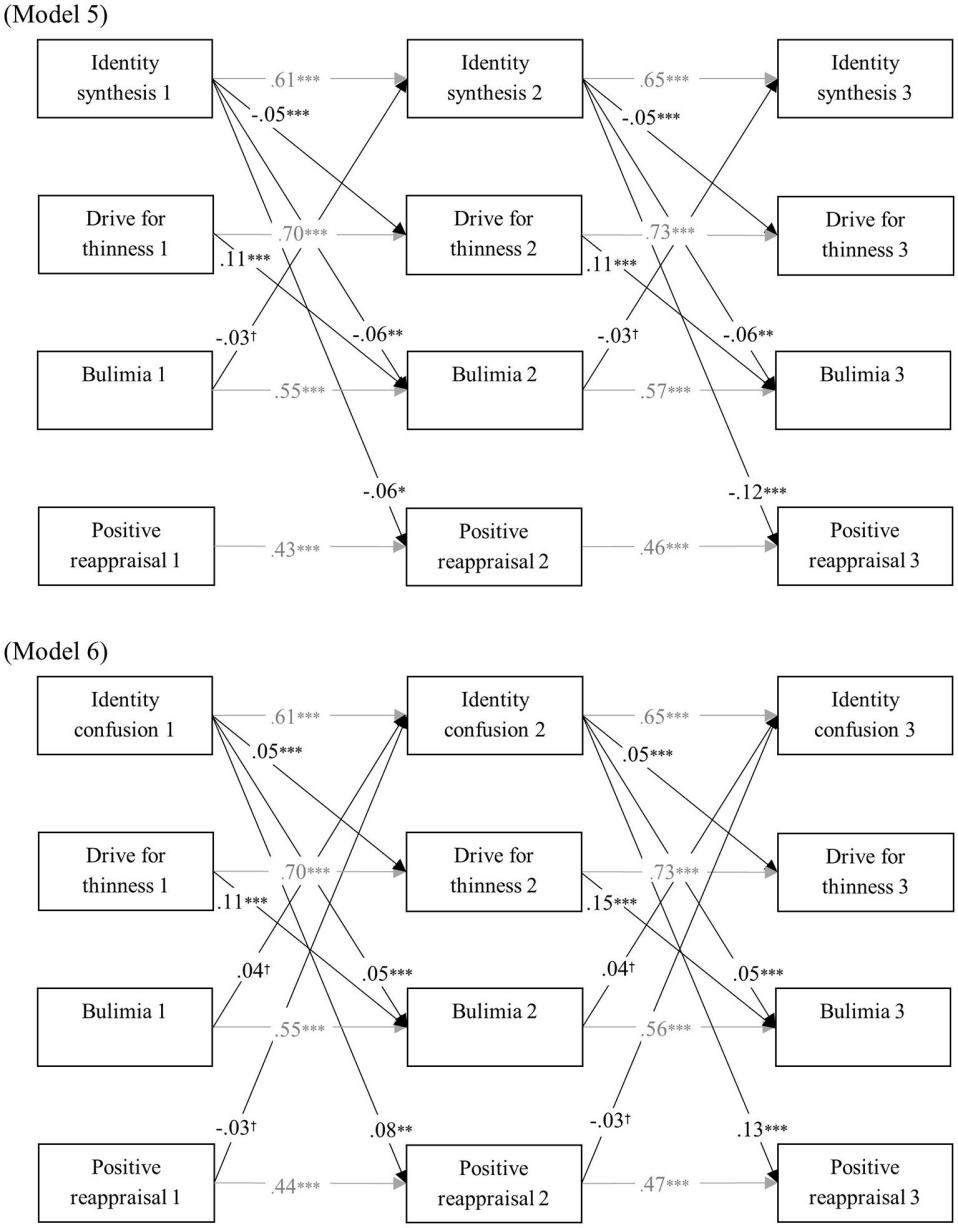
Final cross-lagged models linking identity functioning, ED symptomatology, and positive reappraisal. All models were controlled for age and gender. Within-time associations, associations with age and gender, and insignificant cross-lagged paths were not shown for reasons of clarity. All path coefficients are standardized. ^*†*^*p* < 0.05, ^**^p < 0.01, ^***^p < 0.001.

Table 3 represents model fit of all cross-lagged models. All baseline models had a reasonable fit. Moreover, model comparison indicated that the cross-lagged coefficients were time invariant. The final cross-lagged models are presented in Figures 2–4. In all models, identity synthesis predicted a relative decrease in ED symptomatology, rumination, and catastrophizing over time, and predicted a relative increase in positive reappraisal over time. Similarly, identity confusion predicted a relative increase in ED symptomatology, rumination, and catastrophizing over time, and predicted a relative decrease in positive reappraisal over time. With regard to the predictive value of the cognitive emotion regulation dimensions, different results were found. Although higher levels of catastrophizing and rumination predicted a relative decrease in identity synthesis and a relative increase in identity confusion and ED symptomatology, no such relations were found with positive reappraisal. Additionally, bulimia predicted a relative increase in rumination and catastrophizing over time, whereas no such prediction was found for positive reappraisal. Finally, drive for thinness predicted a relative increase in bulimia in all models.

**Table 3:** Fit indices for Cross-lagged Models at Baseline, Fixed across Time, and Fixed across Gender.

	**S-Bχ2 (*df*)**	***p***	**ΔS-Bχ2 (*df*)**	***p***	**CFI**	**ΔCFI**	**SRMR**	**ΔSRMR**	**RMSEA [90% CI]**	**ΔRMSEA**
**Model 1**										
Baseline	263.545 (16)	<0.001	–	–	0.977	–	0.021	–	0.085 [0.076, −0.094]	–
Fixed across time	279.953 (28)	<0.001	12.792 (12)	0.384	0.976	0.001	0.021	0.000	0.065 [0.058, −0.072]	0.020
Fixed across gender	329.159 (68)	<0.001	6.104 (12)	0.911	0.970	0.006	0.028	0.007	0.060 [0.053, −0.066]	0.005
**Model 2**										
Baseline	272.412 (16)	<0.001	–	–	0.976	–	0.021	–	0.086 [0.077, −0.095]	–
Fixed across time	287.206 (28)	<0.001	11.766 (12)	0.465	0.976	0.000	0.022	0.001	0.065 [0.059, −0.072]	0.021
Fixed across gender	340.875 (68)	<0.001	5.772 (12)	0.927	0.969	0.007	0.029	0.007	0.061 [0.055, −0.068]	0.004
**Model 3**										
Baseline	256.818 (16)	<0.001	–	–	0.976	–	0.021	–	0.084 [0.075, −0.093]	–
Fixed across time	270.874 (28)	<0.001	12.127 (12)	0.435	0.976	0.000	0.022	0.001	0.063 [0.057, −0.070]	0.021
Fixed across gender	317.635 (68)	<0.001	10.533 (12)	0.569	0.970	0.006	0.030	0.008	0.058 [0.052, −0.065]	0.005
**Model 4**										
Baseline	266.017 (16)	<0.001	–	–	0.976	–	0.021	–	0.085 [0.076, −0.094]	–
Fixed across time	279.873 (28)	<0.001	11.990 (12)	0.446	0.976	0.000	0.022	0.001	0.065 [0.058, −0.072]	0.020
Fixed across gender	330.659 (68)	<0.001	10.449 (12)	0.684	0.969	0.007	0.030	0.008	0.060 [0.053, −0.066]	0.005
**Model 5**										
Baseline	294.173 (16)	<0.001	–	–	0.973	–	0.025	–	0.090 [0.081, −0.099]	–
Fixed across time	309.897 (28)	<0.001	10.370 (12)	0.584	0.972	0.001	0.026	0.001	0.068 [0.062, −0.075]	0.022
Fixed across gender	365.679 (68)	<0.001	9.217 (12)	0.684	0.965	0.007	0.033	0.007	0.064 [0.057, −0.070]	0.004
**Model 6**										
Baseline	304.812 (16)	<0.001	–	–	0.972	–	0.025	–	0.091 [0.083, −0.101]	–
Fixed across time	321.220 (28)	<0.001	10.972 (12)	0.531	0.971	0.001	0.026	0.001	0.070 [0.063, −0.077]	0.021
Fixed across gender	365.191 (68)	<0.001	9.387 (12)	0.670	0.964	0.007	0.031	0.005	0.072 [0.065, −0.079]	0.002

*S-Bχ^2^, Satorra-Bentler Chi-square; CFI, Comparative Fit Index; SRMR, Standardized Root Mean Square Residual; RMSEA, Root Mean Square Error of Approximation. While some changes in RSMEA (ΔRMSEA) are larger than 0.015, these changes point to an improvement of model fit in the constrained models. Increasing the degrees of freedom have indeed been found to result in smaller RMSEA values (Taasoobshirazi and Wang, 2016)*.

Due to the non-significant cross-lagged paths with positive reappraisal, the proposed indirect paths through positive reappraisal could not reach significance. The indirect effects were significant for identity synthesis at T1 to (1) drive for thinness at Time 3, through rumination (ß = −0.003, *p* < 0.01) and catastrophizing (ß = −0.002, *p* < 0.05) at Time 2 and (2) to bulimia at Time 3, through rumination (ß = −0.004, *p* < 0.01) and catastrophizing (ß = −0.002, *p* < 0.05) at Time 2. With regard to identity confusion at T1, significant indirect effects were found (1) to drive for thinness at Time 3, through rumination (ß = 0.003, *p* < 0.01) and catastrophizing (ß = 0.002, *p* < 0.05) at Time 2 and (2) to bulimia at Time 3, through rumination (ß = 0.004, *p* < 0.01) and catastrophizing (ß = 0.002, *p* < 0.05) at Time 2. Finally, significant reversed indirect paths were found from bulimia at Time 1 to (1) identity synthesis at Time 3, through rumination (ß = −0.002, *p* < 0.05) and catastrophizing (ß = −0.002, *p* < 0.05) at Time 2 and (2) identity confusion at Time 3, through rumination (ß = 0.002, *p* < 0.05) and catastrophizing (ß = 0.002, *p* < 0.05) at Time 2.

### Multigroup Analyses for Gender

Multigroup analyses with the time-invariant models indicated that the cross-lagged coefficients could be constrained across gender. Differences in model fit with the baseline models are presented in Table 3. These results indicate that the cross-lagged paths linking identity functioning, ED symptomatology, and cognitive emotion regulation were not significantly different for adolescent girls and boys.

## Discussion

The present study aimed to contribute to the growing area of eating disorder (ED) research by exploring the temporal interplay between ED symptomatology, cognitive emotion regulation, and identity development in adolescence. Both issues in identity development and cognitive emotion dysregulation have been found to be important risk and maintenance factors of ED symptomatology, but no study thus far has included them both in longitudinal research. Hence, the three-wave longitudinal design of the present study allows for examining the directionality of effects among these variables. The core finding to emerge from this study is the bidirectional relation between identity functioning and bulimia symptoms through maladaptive cognitive emotion regulation strategies, whereas inconsistent results were found for drive for thinness.

### Mean Level Gender Differences

In the entire sample, mean levels of ED symptomatology were similar to previous studies in community adolescent and adult samples (Nyman-Carlsson et al., 2015; Verschueren et al., 2018). As expected, girls generally experienced more drive for thinness than boys, which is consistent with ED literature (e.g., Lewinsohn et al., 2002; Micali et al., 2014; Verschueren et al., 2020b). Girls also reported more bulimic symptoms than boys, which has been inconsistently found in previous studies (Micali et al., 2014). These results are in line with previous research showing that girls are especially vulnerable to internalize the thin ideal, generally promoting disordered eating (Thompson and Stice, 2001; Knauss et al., 2007). Girls also reported higher scores on identity confusion and lower scores on identity synthesis than boys, which is consistent with the findings of Verschueren et al. (2017) in adolescent and emerging adult samples. Also the mean levels of identity functioning were similar to previous research in a high school sample (Verschueren et al., 2018). Finally, in the present sample, mean levels of all three cognitive emotion regulation strategies were somewhat lower than those in previous research in adolescent and adult samples (Garnefski and Kraaij, 2006; Rey Peña and Extremera Pacheco, 2012). Girls scored higher on the maladaptive strategies and scored lower on positive reappraisal than boys. Previous research has indeed indicated that women tend to ruminate more than men (Hilt et al., 2010; Johnson and Whisman, 2013) and experience more catastrophic worry (Danielsson et al., 2013). Interestingly, the higher scores of positive reappraisal in adolescent boys are inconsistent with previous studies in adolescence and emerging adulthood, where girls scored consistently higher on positive reappraisal (Martin and Dahlen, 2005; Kökönyei et al., 2019). It is not entirely clear why different results were found in the present study, but these findings add to the inconsistent gender differences that are found in emotion regulation research (Nolen-Hoeksema and Aldao, 2011; Goossens et al., 2016).

### Within-Time and Temporal Associations Among the Study Variables

Within-time correlations between the study variables offered a first exploration of the main objective of the present study and provided information on how identity functioning, cognitive emotion regulation strategies, and ED symptomatology were concurrently interrelated. In line with theory and empirical studies, a consistent positive association was found between identity confusion, maladaptive cognitive emotion regulation strategies, and ED symptomatology, meaning that individuals who scored higher on one of those variables compared to their peers, generally also scored higher on the other variables compared to their peers. Negative associations were found between identity synthesis and positive reappraisal on the one hand, and between identity confusion, maladaptive strategies, and ED symptomatology on the other hand.

**Figure 5:**
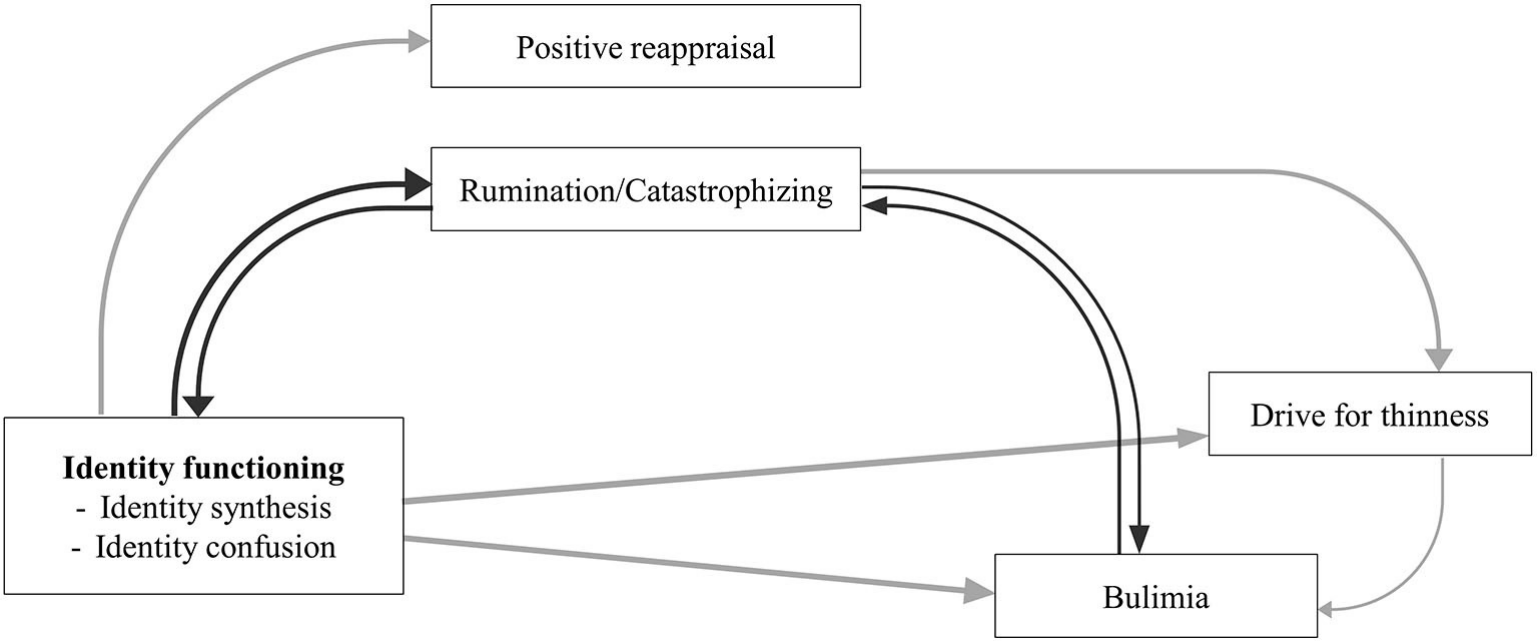
Final model based on the cross-lagged results. Black lines represent bidirectional pathways; gray lines represent unidirectional pathways.

Focusing on the temporal associations between the study variables, cross-lagged path analyses showed somewhat different interconnections. Figure 5 gives a visual representation of the significant cross-lagged paths when combined in one model.

#### From Identity to ED Symptomatology

First of all, direct cross-lagged paths from identity synthesis and identity confusion to drive for thinness and bulimia emerged in the present study (Verschueren et al., 2018). These direct pathways are in line with previous research that have referred to the internalization of beauty ideals, to which identity confused individuals are especially susceptible (Vartanian, 2009; Verstuyf et al., 2014). However, the present study did not investigate the role of this potential mechanism and, hence, results need to be interpreted with caution. This finding does support previous claims on identity functioning being related to body-specific psychopathology such as ED symptomatology (and non-suicidal self-injury; Verschueren et al., 2020a). Interestingly, in DSM-5 Section III (American Psychiatric Association, 2013), an alternative model for personality disorders has been proposed, in which impairments in identity and self-direction are described as important indicators of personality disorders. While the present study does not focus on personality disorders, impairments in identity functioning may have a transdiagnostic value to both personality and body-related psychopathology. In line with other researchers (Klimstra and Denissen, 2017; Kaufman and Crowell, 2018; Verschueren et al., 2020a), further research is necessary to understand how identity functioning may play into general psychopathology.

Interestingly, an indirect pathway was found as well—from identity functioning to ED symptomatology, through maladaptive cognitive emotion regulation strategies (i.e., rumination and catastrophizing). This finding, although needing to be replicated, suggests that individuals who experience identity confusion seem at risk to develop cognitive emotion dysregulation, which in turn may stimulate ED symptomatology. With regard to the identity-emotion regulation paths, some theorists have indeed described how healthy identity functioning is a prerequisite of adaptive emotion regulation (Jørgensen, 2006; Fuchs, 2007), with identity disturbance resulting in a difficulty to regulate emotions and reflect on situations and decisions.

Focusing on the emotion regulation-ED paths—individuals who experience emotion dysregulation may focus on their body shape and engage in eating behaviors as a way to self-regulate, when confronted with intense emotions (Overton et al., 2005). More specifically, in the present study, rumination and catastrophizing predicted a relative increase in both drive for thinness and bulimia symptoms. When ruminating and catastrophizing, individuals fixate on the negative aspects of a certain event, which exacerbates negative affect and cognitions (Garnefski et al., 2001; Nolen-Hoeksema et al., 2008). Hence, individuals who display high levels of rumination and catastrophizing are generally not able to regulate their emotions in an adaptive way, and may engage in binge eating and purging behaviors in an attempt to block or escape from the consistent negative feelings and cognitions (Cooper et al., 2004; Corstorphine, 2006; Nolen-Hoeksema et al., 2007). Disturbed eating have indeed been described as a way to escape the negative cycle between continuous rumination and escalated negative affect (Arbuthnott et al., 2015).

Another possible mechanism may lie in the fact that individuals who score high on rumination and catastrophizing might also experience such negative thoughts when they are confronted with ED-related stimuli. For example, when looking at images from Instagram models or when gaining weight, individuals who have the tendency to ruminate and catastrophize over negative events and emotions, might also be more vulnerable to engage in these kinds of cognitions with regard to their body weight and shape. Over time, this might result in ED thoughts and behaviors (Cowdrey and Park, 2011). However, as we did not assess the content of the ruminative and catastrophizing thoughts in the present study, we cannot verify these assumptions.

#### From ED Symptomatology to Identity

Contrary to expectations, no direct significant cross-lagged pathways from ED symptomatology to identity functioning were found. While previous research in adolescents has shown that bulimia symptoms predicted a relative increase in identity confusion (and a relative decrease in identity synthesis) over time (Verschueren et al., 2018), in the present study, such associations only existed indirectly—through maladaptive cognitive emotion regulation strategies. More specifically, bulimic behaviors predicted a relative increase in both rumination and catastrophizing over time, which in turn predicted a relative increase in identity confusion (and a decrease in identity synthesis). Both binge eating and purging behavior have indeed been described as maladaptive ways to handle intense emotions (Cooper et al., 2004; Corstorphine, 2006), but as they can only temporarily block out intolerable emotions, they are essentially ineffective on the long term (Smyth et al., 2007; Mallorquí-Bagué et al., 2018). Hence, the initial negative thoughts will not only reappear after binge-purging behavior, but may even “rebound” to a much higher frequency. A meta-analysis of Abramowitz et al. (2001) has indeed concluded that thought-suppressing behaviors (such as binge eating and purging) can eventually result in an increase of these thoughts. In line with previous research in female adolescents (Nolen-Hoeksema et al., 2007), our results indicate that engaging in these types of behaviors may stimulate ruminating and catastrophizing thoughts over time.

With regard to drive for thinness, it is important to note that no direct cross-lagged paths to the cognitive emotion regulation strategies were found in the present study. More specifically, experiencing drive for thinness was not related to an increase in rumination or catastrophizing over time, as opposed to binge eating and purging behaviors. This finding might be related to the fact that emotion regulation research has mainly focused on the behavioral component of restrictive eating (Lafrance Robinson et al., 2014; Haynos et al., 2018), while the wish of being thinner and being preoccupied with dieting (i.e., drive for thinness) in itself, might not hold such a strong emotion regulatory function. Hence, while it could be possible that restrictive eating can help regulate emotions (Lafrance Robinson et al., 2014; Haynos et al., 2018), our results indicate that drive for thinness is not prospectively related to a change in the cognitive emotion regulation strategies that were included in the present study.

Finally, direct cross-lagged paths from ruminating and catastrophizing to identity functioning were found—corroborating earlier theorizing that engaging in these maladaptive strategies may result in an inability to make important decisions, a general feeling of emptiness, and the development of identity disturbance (Kernberg, 1975; Linehan, 1993; Jankowski, 2013). The harmful effects of ruminating on important identity questions (i.e., ruminative exploration; Luyckx et al., 2008) have already been demonstrated and related to higher levels of identity confusion and lower levels of identity synthesis (Bogaerts et al., 2018).

#### Positive Reappraisal

Rather unexpectedly, positive reappraisal was not prospectively related to ED symptomatology in the present study. This finding is contrary to previous research that pointed to the protective role of positive reappraisal against binge eating behavior (Nakahara et al., 2000; Danner et al., 2012). However, other studies have found that disturbed eating behavior is not predicted by or related to adaptive cognitive emotion regulation strategies, as they only found significant associations between binge eating and maladaptive strategies (Mills et al., 2015; Goossens et al., 2016). Similarly, it has been consistently reported that psychopathology is generally more strongly associated with maladaptive emotion regulation strategies compared to adaptive strategies (Aldao and Nolen-Hoeksema, 2012). Based on our results, it seems that the ability to give a positive meaning to a certain event does not seem to protect individuals against the development of ED symptoms over time, and the experience of ED symptomatology does not seem to hamper such a positive cognitive ability over time either.

#### No Cross-Lagged Gender Differences

Lastly, although it has been forwarded that especially young women are vulnerable to the dynamic interplay between emotion regulation and ED symptomatology (Overton et al., 2005), multigroup analyses indicated that all direct cross-lagged pathways could be set equal for boys and girls. Mean differences did point to boys generally experiencing less issues with identity functioning, cognitive emotion regulation, and ED symptomatology, but, interestingly, these constructs were interrelated in the same way as they were in girls. This means that, for example, boys with higher scores on identity confusion compared to their peers, would also be more vulnerable to experience an increase in rumination, catastrophizing, and ED symptomatology over time. These findings underscore that similar ED prevention efforts may be used in adolescent boys and girls—targeting identity development and cognitive emotion regulation—as they are related to the development of ED symptomatology in a similar way in both groups.

### Limitations

While the present study made some important contributions to ED research, it was characterized by some limitations. First, cross-lagged panel modeling uses a between-person perspective—focusing on individuals' scores relative to the scores of the sample and on relative change in the constructs over time. Doing so, it cannot distinguish between-person and within-person variance (i.e., how certain processes and mechanisms fluctuate over time within the individual; Orth et al., 2020). Including alternative methods such as random intercepts cross-lagged panel models in future studies may offer information on whether the associations that were found in the present study also exist within an individual. However, most of such alternative methods ideally require more than three measurement points (Orth et al., 2020).

Second, the present study used specific measures of identity functioning (EPSI), cognitive emotion regulation strategies (CERQ), and ED symptomatology (EDI-3). Future studies could explore the interrelations among these study variables using other assessments as well. For example, using a narrative identity approach (McAdams and McLean, 2013), including other emotion regulation dimensions (such as the flexible use of strategies, maintaining behavioral control, and emotional awareness; Gratz and Roemer, 2004) and focusing on a broader range of ED symptomatology (such as excessive exercise, muscular-ideal internalization, and restricted eating; Danielsen et al., 2015; Schaefer et al., 2017) would help to understand the dynamic interplay between these constructs. Similarly, the present study exclusively focused on cognitive emotion regulation strategies and, hence, did not include any behavioral strategies such as seeking social support or distraction (Kraaij and Garnefski, 2019). However, as such cognitive strategies are generally more complex and develop throughout childhood and adolescence (Garnefski et al., 2007), they might not be fully developed in some of the younger participants in the present study. Including behavioral strategies in future research may be more accessible (and protective) to some younger adolescents.

Third, the 1-year interval of the present study does not allow an examination of the assumptions that were made in the present study, regarding the short-term alleviation of intolerable emotions by binge eating, purging, or restrictive eating. For example, studies using the Experience Sampling Method (ESM) might find that right after an individual has engaged in binge eating, they do experience a temporary decrease in ruminative and catastrophizing thoughts. However, as binge eating only temporarily suppresses negative feelings, on the long term, rumination and catastrophizing increase (as was found in the present study).

Fourth, the present study was carried out in high school students and results cannot be generalized to a population of patients with an ED. Including such clinical samples in future studies could possibly replicate the prospective associations among the study variables in a patient sample. Similarly, it would be interesting to investigate the co-development of identity functioning and ED symptomatology throughout an ED treatment and to focus on the predictive value of identity functioning at admission to ED treatment response. Also, while the high school context of the present study minimized dropout due to research taking place during school hours, individuals who had left high school could only participate online. Hence, such online participation resulted in higher dropout in older individuals (de Leeuw and Lugtig, 2014; Verschueren et al., 2018).

Finally, the present study made exclusive use of self-report questionnaires which may cause reporting bias and inflated correlations between the studied variables due to shared method variance. Including alternative methods such as qualitative research or including information of family members may help validating our results.

### Conclusion

In spite of its limitations, the present study is the first empirical investigation into the temporal interplay of identity functioning, cognitive emotion regulation, and ED symptomatology in adolescence, and may have a number of important implications for future practice. As the present findings indicate that identity confusion predicts an increase in the development of ED symptomatology over time, ED prevention programs should target a healthy identity development in which individuals learn to develop other sources of self-esteem than their appearance and body image (Corning and Heibel, 2016). Individuals could learn to integrate various self-aspects (both positive and negative) to experience oneself as a coherent whole. Furthermore, with the help of a therapist or teacher, they can explore personal interests, values, and talents—indicative of identity synthesis (Erikson, 1968). Similarly, it is crucial to learn adolescents to handle negative emotions and events in an adaptive way, as results showed that rumination and catastrophizing predict an increase in ED symptomatology over time. In youth who present with ED symptoms, it might be helpful to explore when and why the individual engages in binge eating or purging behavior. In youth who present with ED symptoms, it might be helpful to explore when and why the individual engages in binge eating or purging behavior. Learning to identify the negative affect and the accompanied ruminative and catastrophizing thoughts, might help the individual to use other emotion regulation strategies than engaging in bulimic behaviors (such as problem focused behavior, acceptance, and positive reappraisal). Finally, especially bulimic behavior seems to hamper identity functioning through the development of maladaptive cognitive emotion regulation strategies. More specifically, behavioral programs (such as exposure therapy) that target both binge eating and purging behaviors in adolescents, might also result in decreases in both rumination and catastrophizing thoughts, which would in turn be associated with greater identity synthesis over time. Again, in youth who present with ED symptoms, intervention purposes may be especially helpful when they focus on the emotion regulatory function of bulimic behavior.

## Data Availability Statement

The raw data supporting the conclusions of this article will be made available by the authors, without undue reservation.

## Ethics Statement

The studies involving human participants were reviewed and approved by Social and Societal Ethics Committee of KU Leuven. Written informed consent to participate in this study was provided by the participants' legal guardian/next of kin.

## Author Contributions

TB, LC, and KL oversaw the data collection. MV, NP, and LR helped with the data collection. MV, KL, and LC conceived of the presented idea, constructed the hypotheses, analyzed the data, and interpreted the results. MV wrote the manuscript with critical revision from all authors (KL, LC, NP, LR, TB, and PM).

## Conflict of Interest

The authors declare that the research was conducted in the absence of any commercial or financial relationships that could be construed as a potential conflict of interest.

## Footnotes

^1^In order to compare the mean levels of the entire sample to previous studies in community samples, the traditional mean scale scores are represented. When using the untransformed mean scale scores instead of the factor scores, drive for thinness ranged from 2.26 to 2.32 and bulimia ranged from 1.90 to 1.92 on all time points. With regard to identity functioning, the mean scale scores of identity synthesis ranged from 3.62 to 3.65 and those of identity confusion ranged from 2.59 to 2.63 on all time points. Finally, mean scale scores of rumination ranged from 2.75 to 2.76, those of catastrophizing ranged from 2.04 to 2.12, and those of positive reappraisal ranged from 3.18 to 3.27 on all time points.
