# Supplementary material for: Identity Functioning and Eating Disorder Symptomatology: The Role of Cognitive Emotion Regulation Strategies
Source: Front Psychol. 2021 May 28;12:667235. doi: 10.3389/fpsyg.2021.667235 (PMC8194491; doi:10.3389/fpsyg.2021.667235)
Supplement: Supplementary file 1 [file Table_1.docx]

**Supplementary Material**

Table 1. *Fit Indices for Testing Longitudinal Measurement Invariance.*

| Model | S-Bχ² (*df*) | *p* | ΔS-Bχ² (*df*) | *p* | CFI | ΔCFI | TLI | ΔTLI | SRMR | ΔSRMR | RMSEA [90% CI] | ΔRMSEA |
| --- | --- | --- | --- | --- | --- | --- | --- | --- | --- | --- | --- | --- |
| ***EDI-3*** | | | | | | | | | | | | |
| Configural | 2566.597 (225) | < .001 | - | - | .914 | - | .895 | - | .086 | - | .073 [.071 - .076] | - |
| Metric | 2607.096 (253) | < .001 | 72.3357 (28) | < .001 | .913 | .001 | .906 | .011 | .091 | .005 | .069 [.067 - .072] | .004 |
| **Scalar** | **2852.129 (281)** | **< .001** | **231.8961 (28)** | **< .001** | **.905** | **.008** | **.908** | **.002** | **.093** | **.002** | **.069 [.066 - .071]** | **.000** |
| Full uniqueness | 3197.244 (309) | < .001 | 320.6280 (28) | < .001 | .894 | .011 | .906 | .002 | .099 | .006 | .069 [.067 - .072] | .000 |
| ***EPSI*** |  |  |  |  |  |  |  |  |  |  |  |  |
| Configural | 1567.810 (159) | < .001 | - | - | .918 | - | .898 | - | .039 | - | .068 [.064 - .071] | - |
| Metric | 1688.025 (183) | < .001 | 110.0828 (24) | < .001 | .912 | .006 | .905 | .007 | .053 | .014 | .065 [.062 - .068] | .003 |
| Scalar | 1826.696 (207) | < .001 | 124.9730 (24) | < .001 | .906 | .006 | .910 | .005 | .056 | .003 | .063 [.061 - .066] | .002 |
| **Full uniqueness** | **2000.526 (231)** | **< .001** | **176.5823 (24)** | **< .001** | **.897** | **.009** | **.912** | **.002** | **.064** | **.008** | **.063 [.060 - .065]** | **.000** |
| ***CERQ*** | | | | | | | | | | | | |
| Configural | 1570.767 (297) | < .001 | - | - | .954 | - | .928 | - | .034 | - | .047 [.045 - .050] | - |
| Metric | 1615.313 (333) | < .001 | 32.8255 (36) | .620 | .953 | .001 | .936 | .008 | .036 | .002 | .045 [.043 - .047] | .002 |
| Scalar | 1761.010 (369) | < .001 | 141.6818 (36) | < .001 | .949 | .004 | .937 | .001 | .037 | .001 | .044 [.042 - .046] | .001 |
| **Full uniqueness** | **1884.179 (405)** | **< .001** | **126.9719 (36)** | **< .001** | **.946** | **.003** | **.939** | **.002** | **.038** | **.001** | **.044 [.042 - .046]** | **.000** |

*Note.* S-Bχ² = Satorra-Bentler chi-square; CFI = Comparative Fit Index; TLI = Tucker-Lewis Index; SRMR = Standardized Root Mean Square Residual; RMSEA = Root Mean Square Error of Approximation; EDI-3 = Eating Disorder Inventory-3; EPSI = Erikson Psychosocial Stage Inventory; CERQ = Cognitive Emotion Regulation Questionnaire.
